# Tetravalent recombinant dengue virus-like particles as potential vaccine candidates: immunological properties

**DOI:** 10.1186/s12866-014-0233-3

**Published:** 2014-12-18

**Authors:** Yan Liu, Junmei Zhou, Zhizhun Yu, Danyun Fang, Chunyun Fu, Xun Zhu, Zhenjian He, Huijun Yan, Lifang Jiang

**Affiliations:** Department of Microbiology, Zhongshan School of Medicine, Sun Yat-Sen University, Guangzhou, Guangdong 510080 People’s Republic of China; Key laboratory of Tropical Disease Control (Sun Yat-sen University), Ministry of Education, 74 Zhongshan Road 2, Guangzhou, 510080 People’s Republic of China

**Keywords:** Dengue virus, Virus-like particles, *Pichia pastoris*, Vaccine, Immunological properties

## Abstract

**Background:**

Currently, a licensed vaccine for Dengue Virus (DENV) is not yet available. Virus-like particles (VLP) have shown considerable promise for use as vaccines and have many advantages compared to many other types of viral vaccines. VLPs have been found to have high immunogenic potencies, providing protection against various pathogens.

**Results:**

In the current study, four DENV-VLP serotypes were successfully expressed in *Pichia pastoris*, based on co-expression of the prM and E proteins. The effects of a tetravalent VLP vaccine were also examined. Immunization with purified, recombinant, tetravalent DENV1-4 VLPs induced specific antibodies against all DENV1-4 antigens in mice. The antibody titers were higher after immunization with the tetravalent VLP vaccine compared to titers after immunization with any of the dengue serotype VLPs alone. Indirect immunofluorescence assay (IFA) results indicated that sera from VLP immunized mice recognized the native viral antigens. TNF-α and IL-10 were significantly higher in mice immunized with tetravalent DENV-VLP compared to those mice received PBS. The tetravalent VLP appeared to stimulate neutralizing antibodies against each viral serotype, as shown by PRNT_50_ analysis (1:32 against DENV1 and 2, and 1:16 against DENV3 and 4). The highest titers with the tetravalent VLP vaccine were still a little lower than the monovalent VLP against the corresponding serotype. The protection rates of tetravalent DENV-VLP immune sera against challenges with DENV1 to 4 serotypes in suckling mice were 77, 92, 100, and 100%, respectively, indicating greater protective efficacy compared with monovalent immune sera.

**Conclusions:**

Our results provide an important basis for the development of the dengue VLP as a promising non-infectious candidate vaccine for dengue infection.

## Background

Dengue virus (DENV), a mosquito-borne RNA virus, belonged to the genus Flavivirus of the family *Flaviviridae*, is the cause of a range of well described clinical diseases, dengue fever (DF), dengue shock syndrome (DSS) and dengue hemorrhagic fever (DHF) [1,2]. Globally, over 2.5 billion people are at risk for DENV infection in tropical and subtropical countries and regions, and approximately 50 to 100 million new cases of dengue infection and 500,000 cases of DHF and/or DSS occur annually [3]. Prevention and control of widespread dengue infection has been a priority of the World Health Organization (WHO) for three decades; however, a licensed vaccine for DENV is not yet available [4].

There are four antigenically distinct serotypes of DENV, designated as DENV1-4. Dengue fever can be caused by any one of these four serotypes and life-long immunity against a distinct serotype can be established after infection. However, the severe forms of DENV infection, DHF and DSS, often occur when individuals are infected a second time by a different serotype [5-7]. The increased severity of subsequent infections is believed to result, at least in part, from antibody-dependent enhancement (ADE) of DENV infection, in which antibody-mediated neutralization does not occur. Instead, virus-antibody complexes facilitate viral entry into cells that express the Fcγ receptor, such as monocytes and macrophages [8-10]. For these reasons, an effective dengue vaccine should be able to induce long-lived protective immunity simultaneously against all four DENV serotypes.

Three structural proteins (capsid (C), premembrane/membrane (prM/M) and envelope (E)) and seven nonstructural proteins (NS1, NS2a, NS2b, NS3, NS4a, NS4b and NS5) are encoded in the RNA genome of the dengue virus. The C protein forms the main structural component of the nucleocapsid [11] and the formation of M, from prM, appears to be the crucial, terminal event in virion morphogenesis [12,13]. The E protein is located on the surface of mature dengue virions and is the principal glycosylated structural protein (MW 51,000 to 60,000). The E protein mediates viral entry and induces a protective immune response [14-16]. The antibody response to DENV infection is mainly directed against the E and prM glycoproteins that are present on the virion surface [17-19]. Antibodies have been found to both neutralize and enhance DENV infectivity, *in vivo* and *in vitro*, and thus appear to play a dual role in controlling DENV infection [10,20-24]. Presumably, this adverse association might predict a potential risk for development of DHF or DSS in an individual who had previously been vaccinated with a specific DENV serotype and then is subsequently infected with a different serotype. Indeed, the majority of DENV vaccines under development are multivalent and aimed at producing immunity potent enough to protect against all four DENV serotypes [25].

Recent advances in genetic manipulation and vaccinology have lead to renewed hope for the development of live attenuated vaccines, subunit vaccines, DNA vaccines and viral-vector vaccines. Of particular interest is the effort to develop recombinant subunit vaccines, based on the viral proteins, capable of mimicking the overall structure of viral particles and inducing an optimal immune response. To this end, the virus-like particles (VLP) approach appears promising and advantageous over many other structural forms of vaccines. VLPs have been found to provide high immunogenic potency in protecting against various pathogens, such as human papillomavirus [26].

It has been demonstrated that co-expression of flavivirus prM and E, or alternatively, C, prM, and E led to production of VLPs that were composed of spherical membrane vesicles containing prM/M and E embedded in a lipid bilayer with or without a nucleocapsid, similar to the morphology of natural virions. These VLPs showed immunogenicity and were able to elicit neutralizing antibodies and virus-specific cytotoxic T lymphocytes [27-29]. Our previous studies demonstrated that co-expression of the prM and E proteins of DENV1, DENV2, and DENV3 produced antigenic DENV-VLP [30-32]. In the current study, DENV4-VLP was constructed and expressed, and the immunological properties and protection level of tetravalent DENV-VLP were evaluated in mice challenged with all four DENV serotypes. The data suggest that antibody responses and cellular immunity induced by monovalent and tetravalent DENV-VLP were comparable. Tetravalent DENV-VLP had better protective efficacy compared to monovalent VLP in suckling mice. The results indicate that tetravalent DENV-VLP could be a promising vaccine candidate.

## Methods

### Cells and viruses

C6/36 *Aedes albopictus* (ATCCNo.CRL-1660) was cultured at 28°C in MEM (Gibco, Guangzhou, China) supplemented with 0.11% of sodium bicarbonate and 10% newborn calf serum (Gibico, USA). BHK-21 *Mesocricetus auratus* was cultured in DMEM (Gibico, Guangzhou, China) supplemented with 10% fetal bovine serum (Gibco, USA) at 37°C in 5% CO_2_. Each dengue virus serotype was passaged and propagated in C6/36 cells and titrated in BHK-21 cells. The DENV1 strain, GZ01/95 (GenBank accession No. EF032590), and DENV2 strain, ZS01/01 (GenBank accession no. EF051521), had been previously isolated, sequence-verified and preserved in our laboratory. DENV3 strain H87 and DENV4 strain H241 were supplied by the Institute for Viral Disease Control and Prevention, China CDC. The viral titer was determined after removal of cell debris via centrifugation. The virions used for mice immunization were inactivated with 1:2000 β-propionolactone and the viron concentration was subsequently detected using the BCA method (Biocolor, Shanghai, China).

### Construction of DENV-VLP expression plasmids

The *P. pastois* host strain, *X33* (Invitrogen, Guangzhou, China), and the expression vector, pGAPZαA (Invitrogen), have been previously described in detail [30,31]. The cDNA of virions of each DENV serotype was obtained by RT-PCR and the genes coding for the prM and E proteins were amplified. The amplified prM-E genes were subsequently linearized and ligated into the pGAPZαA (Invitrogen) vectors in frame with the α-factor secretion signal (for DENV1/2-VLP expression) or the signal peptide of prM (for DENV3/4-VLP espression). The recombinant plasmids for expressing DENV1-4 VLP were named pGAPZα-prME-D1, pGAPZα-prME-D2, pGAPZ-sprM/E-D3, and pGAPZ-sprM/E-D4.

### Expression and purification of DENV-VLP

Expression and purification of DENV-VLP was done as previously described [30-32]. Briefly, the four recombinant plasmids were electroporated into the *Pichia pastoris* host strain, *X33*. The yeast cells were then harvested and disrupted with glass beads in breaking buffer (50 mmol/L sodium phosphate, pH 7.4; 1 mmol/L ethylene diamine tetraacetic acid (EDTA); 1 mmol/L phenylmethyl sulfonylfluoride (PMSF); and 5% glycerol). The lysates were subjected to ultracetrifugation at 153 000 × g for 6 hours at 4°C (HATICHI, P80AT rotor, Ibaraki Prefecture, Japan) using a 5 ~ 50% sucrose density gradient before the sucrose fractions were collected and analyzed via Western blot, using the MAb 15 F3-1 (ATCC, USA), 3H5-1 (ATCC, USA), 5D4-11 (ATCC, USA), and IH10-6 (ATCC, USA) to detect E proteins expression for each DENV-VLP serotype. The formation of VLP was demonstrated using electron microscopy. Finally, protein concentration was assessed using the BCA method (Biocolor, Shanghai, China).

### Virus titer assay

Virus quantitation was determined using a standard plaque assay as previously described [30,31]. Briefly, 0.25 mL of DENV dilutions (10-fold) was added to duplicate wells of BHK-21 cells, which were cultured overnight in 24-well plates before the media was removed. The plates were incubated for 2 h after which the media was aspirated and replaced with 0.8 mL of 0.8% methyl-cellulose medium (with 4% newborn calf serum). The plates were then incubated for 7 days, the media was removed and the cells were fixed in 4% formaldehyde and stained with crystal violet. Plaques were counted visually and the concentrations of plaque-forming units per mL (PFU/mL) were calculated.

### Mice immunization

Experiments with mice were conducted in compliance with a protocol approved by the Institutional Animal Care and Use Committee of Sun Yat-sent University based on the Ethical Principles in Animal Experimentation. Specific pathogen-free female BALB/c mice, aged three to four weeks, were supplied by the Experimental Animal Center of Sun Yat-sen University (Guangzhou, China) and were divided into ten groups according to immunogen. The mice were inoculated intraperitoneally (i.p.) with monovalent DENV-VLP (25 μg per dose and in 15 mice (n = 15)) of a specific serotype or a tetravalent combination (25 μg of each serotype per dose and in 30 mice (n = 30)). Freund’s complete adjuvant (Sigma) was used for priming and Freund’s incomplete adjuvant was used for boosters. Booters were twice at an interval of 2 weeks. Equal amounts of PBS (n = 30) or inactivated DENV virions (25 μg per dose and in 15 mice (n = 15)) were used as controls. Blood samples were collected on days 0, 14 and 28, via the tail vein, for measurement of serum IgG. Seven days after the last inoculation, 1/3 of the mice in each group were euthanized. The spleen of each mouse was removed and splenocytes were isolated in order to test cytokine profiles and serum was collected for further immunological analysis.

### Enzyme-linked immunosorbent assay

Antigen specific serum IgG antibodies were titered using an amplified sandwich ELISA system. Briefly, 96 wells polystyrene plates (Costar, USA) were coated over night at 4°C with 100 μL/well of 5 ng/ml inactivated dengue virus antigen or VLP antigen and then blocked in coating buffer containing 5% fat-free milk powder for 1 h at 37°C. The plates were then incubated with 100 μl/well of sera from each group, along with a 2-fold serial dilution of PBS-T (starting from 1:100), at 37°C for 1 h. Bound IgG was detected using goat anti-mouse IgG-peroxidase conjugate (Santa Cruz, USA). A volume of 3,3′, 5,5′-tetramethly benzidine (TMB) substrate was then added and plates were incubated at 37°C for 15 min. The reaction was stopped with 50 μl of 2 M H_2_SO_4_. Absorbance was measured at 450 nm using an automated ELISA reader (EL×800 BioTek). An absorbance value two-fold above the mean pre-vaccine serum value plus two standard deviations was considered to be a positive result.

### Immunofluorescence assay

C6/36 cells were passaged in MEM medium containing 10% fatal bovine serum (FBS) and used to be infected with 100 PFU of each DENV serotype. Cells were harvested 2 ~ 4 days post-infection, resuspended in MEM containing 10% newborn calf serum, dropped onto slides and incubated at 37°C for 8 h. The slides were then fixed with acetone at −20°C for 15 min. Antisera diluted 1:80, was then added to the slides and the slides were incubated at 37°C for 1 h. Normal mouse sera, diluted 1:80, was used as the negative control. Lastly, fluorescein isothiocyanate (FITC)-conjugated goat anti-mouse IgG was added and the slides were incubated at 37°C for 45 min. Positive cells were detected using a fluorescent microscope.

### Cytokines ELISPOT assay

The cytokines, IFN-γ, TNF-α, and IL-10, from immunized mice were measured using ELISPOT kits (Ucytech, The Netherlands), according to the manufacturer’s instructions. Briefly, ELISPOT 96-well plates (Millipore, USA) were coated with 100 μl of anti-mouse IFN-γ, anti-mouse IL-10, or anti-mouse TNF-α (5 μg/ml in coating buffer). The plates were washed twice and blocked with blocking solution for 2 h. A volume of 100 μL freshly isolated splenocytes (2 × 10^5^ cells) from the immunized mice were subsequently transferred to each well and stimulated with dengue virus at 37°C for 24 h. The cells were then washed away, and a secondary biotinylated anti-cytokine mAb was added to each well, followed by a streptavidin-HRP and AEC substrate solution system. Finally, the spots were counted using an ImmunoSpot® Analyzer (Cellular Technology Ltd. USA).

### Antibody neutralization assay

The neutralizing ability of antibodies was measured using a 50% plaque reduction neutralization test (PRNT_50_). Briefly, BHK-21 cells were grown to 80% confluence in 24-well plates and infected with 200 μL DENV1 to 4 (150 ~ 200 PFU/mL). The plates were then pre-incubated with two-fold serial dilutions of mouse serum (1:4 ~ 1:64) at 37°C for 1 h. The virus-serum mixture was aspirated, added along with 0.8 mL overlay medium, and incubated at 37°C with 5% CO_2_ for 7 days. Dengue virus plaques were counted by naked eye or by scanning the cluster plate into Adobe Photoshop CS for further magnification. PRNT_50_ titers are defined as the maximal serum dilution that inhibits 50% of plaque formation compared to the number of plaques in infected cell wells with no sera.

### Suckling mice passive protection assay

Newborn (1 day old) BALB/c mice were purchased from the Experimental Animal Center of Sun Yat-sen University (Guangzhou, China) and were divided into twelve groups. The 1:10 sera dilution was incubated with each DENV serotype (400PFU) at 37°C for 1 h. Suckling mice were injected intracerebrally with 20 μL of sera-virus mixture. Morbidity (paralysis, ruffling, slowing of activity, kyphoscoliosis and death) and/or mortality were recorded daily, for 3 weeks, post-challenge in all mice.

### Statistical analysis

Data were analyzed for statistical significance using SPSS software (version 13.0). Statistical comparisons among groups were analyzed by one way ANOVA. Kaplan-Meier survival curves used for survival analysis and were analyzed by the log rank test.

## Results

### Expression and purification of DENV-VLP

**Figure 1:**
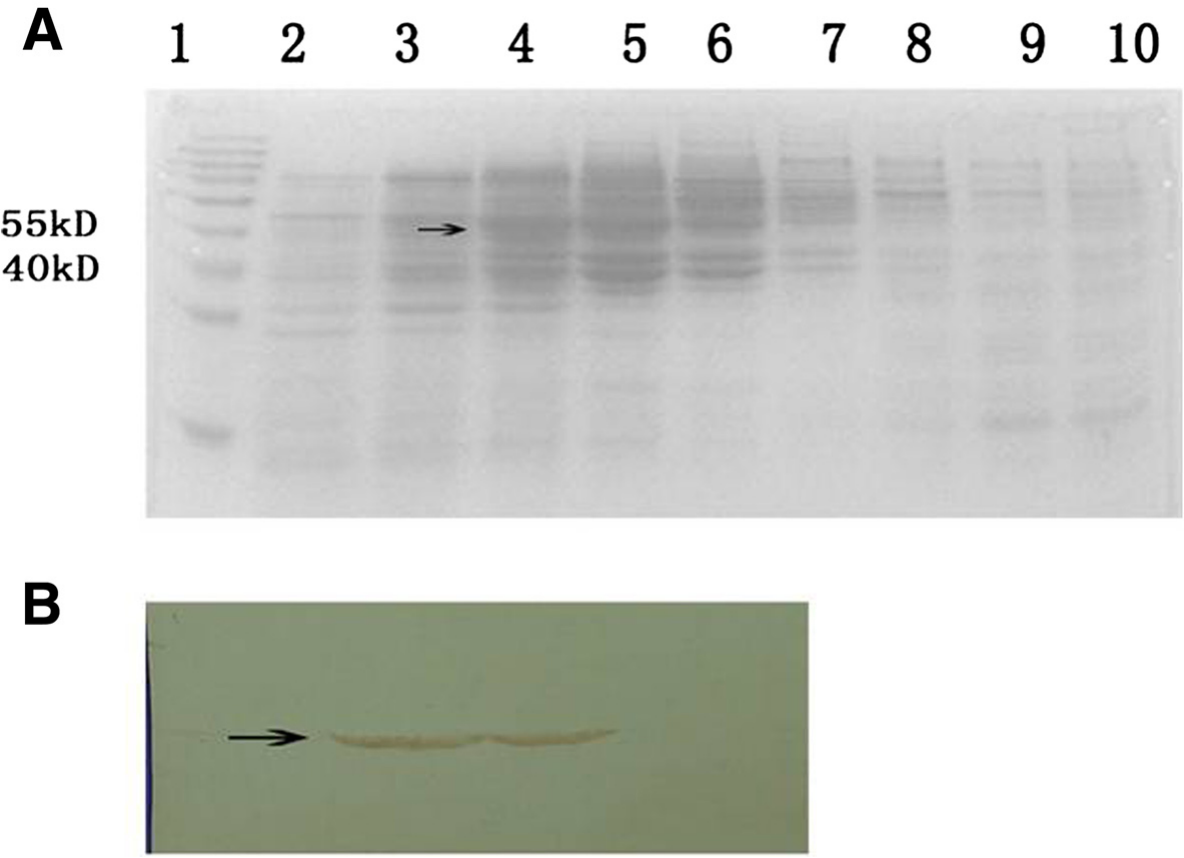
**Secretion analysis of DENV4 VLPs. (A)** SDS-PAGE analysis of different fractions from sucrose density gradient ultracentrifugation. Sucrose gradient sedimentation and SDS-PAGE analysis of DENV4 VLP secretions. The pGAPZaA-sprME-D4-X33 yeast lysates were centrifuged by sucrose density gradient ultracentrifugation and different fractions were collected and analyzed by SDS-PAGE. 1: Marker; 2: 10%; 3: 15%; 4: 20%; 5: 25%; 6: 30%; 7: 35%; 8: 40%; 9: 45%; 10: 50%. **(B)** Western Blot analysis of E protein expression. The 20 ~ 25% fractions collected from sucrose density gradient ultracentrifugation were analyzed for E protein expression using the MAb IH10-6 (anti-DENV4 E protein). Arrows indicate E antigen. The size of the molecular weight marker is shown in kDa.

Yeast lysates were analyzed for expression of E proteins by Western blot using mAbs as described in Methods. A ~50 kDa recombinant E protein band was detected in the lysates of each DENV-VLP expression clones [30-32] (Figure 1A). The lysates of yeast cells expressing DENV1 to 4 VLPs were analyzed using a 5 ~ 50% sucrose density gradient centrifugation. Western blot results indicated that DENV-VLP was present in the faction 20% ~ 25% [30-32] (Figure 1B). The spherical particles with diameters of about 30 nm were detected by electron microsopy [30-32] (data not shown). After the fractions containing VLP were harvested and mixed, the recombinant protein concentration was adjusted to 0.5 mg/mL, then aliquoted and stored −80°C.

### Immunogenicity of purified tetravalent DENV-VLP

**Figure 2:**
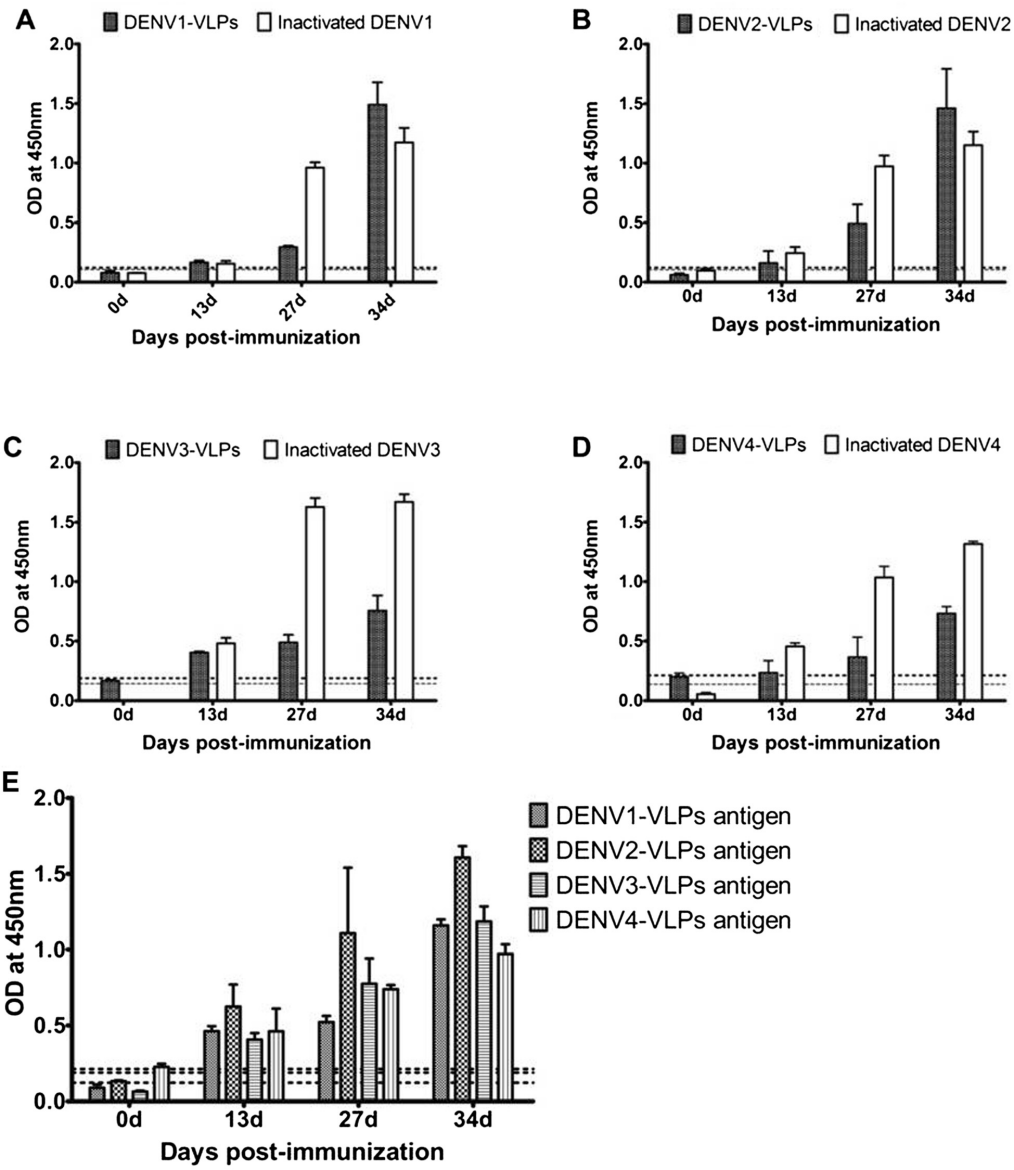
**Indirect ELISA analysis of Dengue Virus or VLP specific IgG antibody.** BALB/c mice were intraperitoneally immunized with 25 μg monovalent DENV1-4 VLP, DENV1-4 virions, or tetravalent combination (25 μg of each serotype) a total of three times at two-week intervals. Sera were collected on days 0, 13, 27, and 34 and indirect ELISA was used to test for antigen specific IgG. Sera from monovalent VLP groups and the tetravalent VLP group reacted with DENV1-4 VLP antigens and sera from inactivated DENV groups reacted with dengue virus 1–4 antigens. Data are expressed as mean absorbance at OD450 nm with a standard error of the mean (SEM) bar. The baseline (dashed line) indicates negative values with normal mouse serum. **A**. Binding of sera from mice immunized with DENV1-VLP and inactivated DENV1 virions to respective antigens; **B**. Binding of sera from mice immunized with DENV2-VLP and inactivated DENV2 virions to respective antigens; **C**. Binding of sera from mice immunized with DENV3-VLP and inactivated DENV3 virions to respective antigens; **D**. Binding of sera from mice immunized with DENV4-VLP and inactivated DENV4 virions to respective antigens; **0045**. Binding of sera from mice immunized with tetravalent VLP to respective antigens.

**Figure 3:**
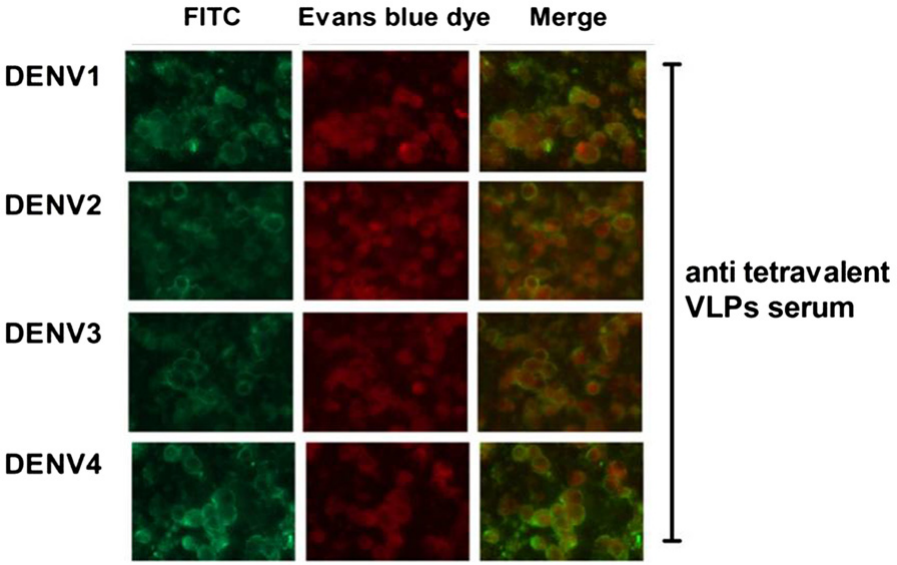
**Indirect immunofluorescence detected that sera from tetravalent VLPs recognized DENV antigens.** The C6/36 cells infected with DENV1-4 were fixed, incubated with a 1:40 dilution of antisera from the tetravalent VLP group and then stained for the virus (green). Evens blue dye staining was used to label cells (red). Magnification 400 × .

Purified, recombinant, tetravalent DENV1-4 VLP antigen was tested for the ability to induce specific antibodies against each DENV1-4 VLP antigen in mice via ELISA. Antibody titers were higher after immunization with tetravalent DENV VLP compared to those after immunization with any of the the monovalent serotype dengue VLPs (Figure 2). Indirect immunofluorescence assay (IFA) results showed that serum from mice immunized with either monovalent or tetravalent VLPs recognized homologous viral antigens (Figure 3). The antibody titers ranged from 1:320 to 1:640 and the titers from the group immunized with tetravalent VLPs were higher than any of the corresponding antibody titers from the groups immunized with monovalent VLPs. These results indicate that immunization with tetravalent VLPs induced stronger antibody responses than immunized with monovalent VLPs.

### Cytokine production measured by ELISPOT

Spleen cells were isolated on day 34 (7 days after the last immunization) to assess the cellular immune response to tetravalent DENV VLP. IFN-γ, TNF-α, and IL-10 cytokine secretion was analyzed, via ELISPOT, following their *ex vivo* stimulation of the cells with inactivated DENV1-4 virions.

**Figure 4:**
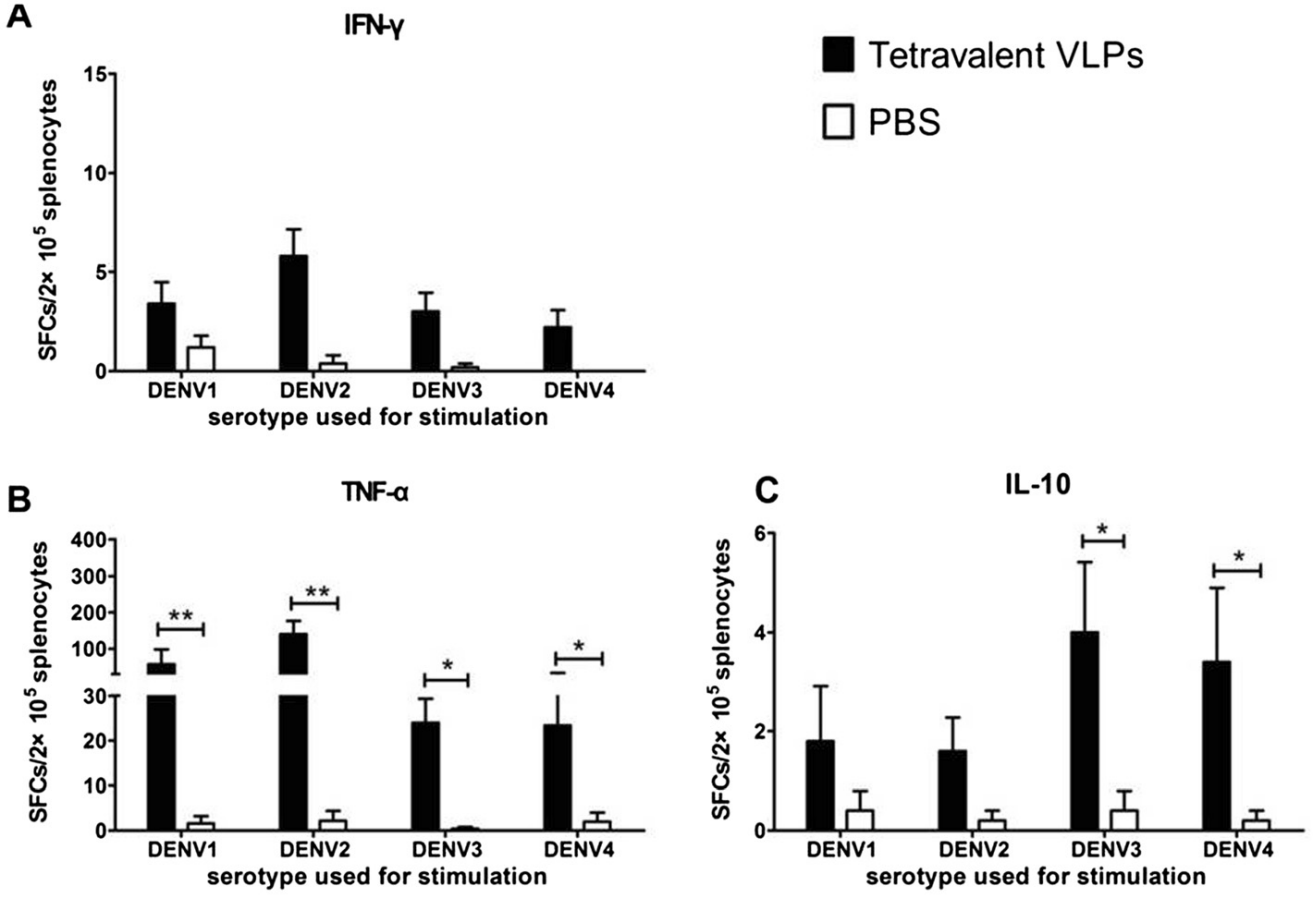
**ELISPOT assay.** The mice immunized with tetravalent DENV-VLP were euthanized 7 days after the 3rd immunization and the spleen cells were isolated and stimulated *in vitro* with inactivated virions of each DENV type. IFN-γ **(A)**, TNF-α **(B)**, and IL-10 **(C)** producing lymphocytes were enumerated by ELISPOT assay. The mean number of spot forming cells (SFCs)/2 × 10^5^ (splenocytes) is shown as virions-stimulated with an SEM bar. *indicates statistical significance (*P < 0.05; **P < 0.01).

As shown in Figure 4, there was no significant difference in the number of splenocytes secreting IFN-γ from animals immunized with tetravalent DENV VLP compared to PBS control, after stimulation with all four dengue serotype virions. The number of splenocytes secreting TNF-α was higher in the tetravalent DENV VLP group compared to the control group and the number of splenocytes secreting TNF-α was higher after stimulation with DENV1 or 2 virions than with DENV3 or 4 virions. The overall number of IL-10 secreting cells was not high in tetravalent DENV VLP group, however, the mean number of cells secreting IL-10 was significantly higher in this group after stimulation with DENV3 or 4 virions compared to the PBS control group. Conversely, there was no significant difference in IL-10 secreting cells between the teravalent DENV-VLP and control groups after stimulation with DENV1 or 2 virions.

### Virus neutralizing antibodies induced by DENV-VLP in mice

**Figure 5:**
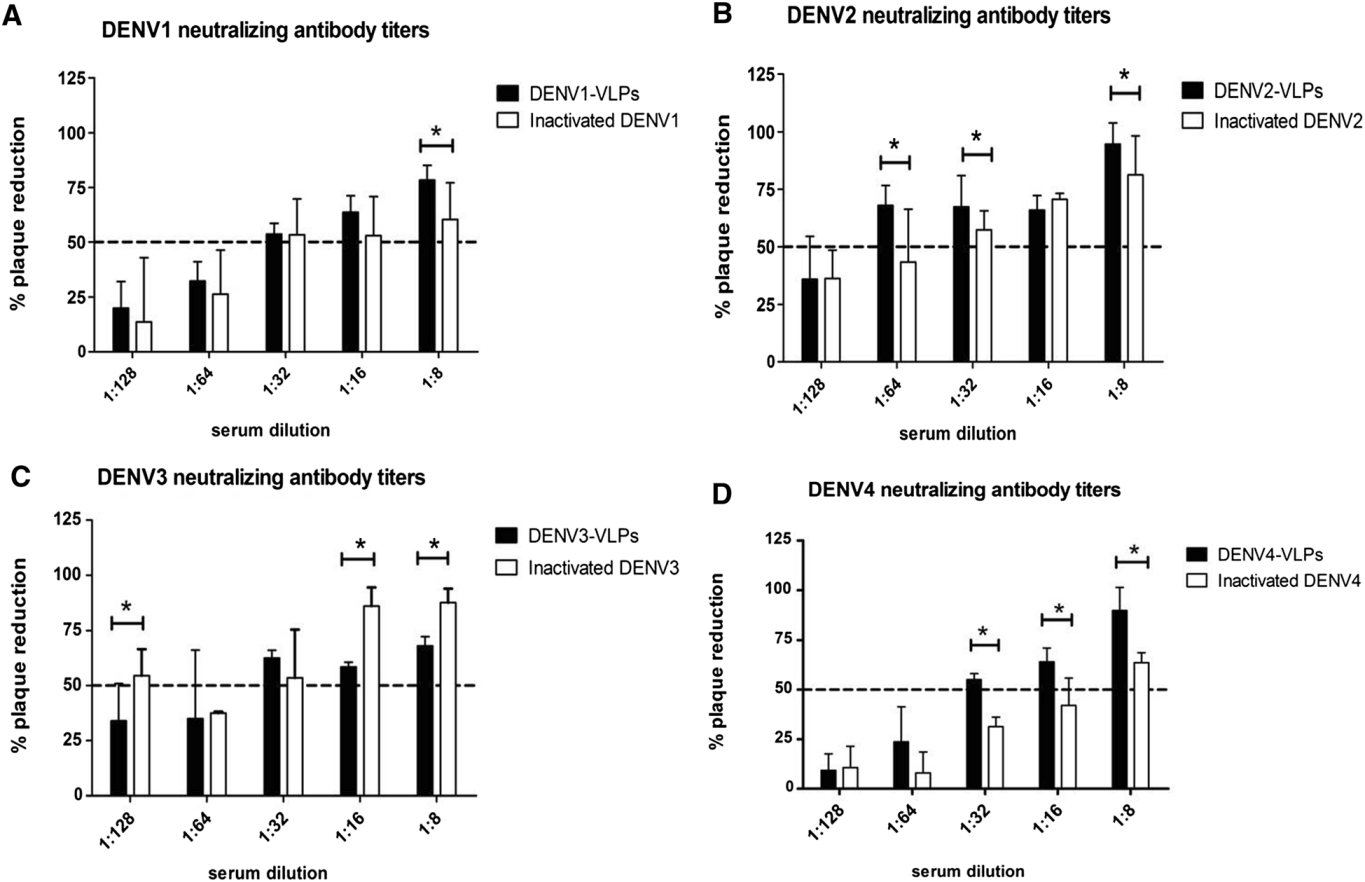
**Detection of monovalent immune serum neutralizing antibody against DENV.** Balb/c mice were immunized with 25 μg monovalent DENV VLP or virions a total of three times at a two week interval. On day 7 after the 3rd immunization, neutralizing antibodies against DENV1 **(A)**, DENV2 **(B)**, DENV3 **(C)**, and DENV4 **(D)** were assessed using a PRNT_50_ assay. 150-200PFU DENV were incubated with serially diluted mouse antisera in 24-well plates, using BHK-21 cell lines. Data from each group is expressed as the mean percentage of plaque reduction with an SD bar (n = 3). The PRNT_50_ titers for each immune sera after immunization with each monovalent vaccine against the corresponding virus were 1:32, 1:64, 1:32, and 1:32, respectively. *indicates statistical significance (*P < 0.05; **P < 0.01).

Virus neutralizing antibody responses to the homologous serotypes were determined by PRNT_50_ analysis of mouse sera in order to evaluate the effective humoral immune response induced by DENV-VLP. The maximum neutralizing antibody titer of the DENV1-VLP group was equivalent to that of the inactivated DENV1 group (Figure 5). The maximum neutralizing antibody titer in the DENV2-VLP group was 1:64, which was higher than the 1:32 titer in the inactivated DENV2 group. Though the maximum titer in both the DENV3-VLP and inactivated DENV3 groups was 1:32, the% plaque reduction was slightly lower in the DENV3-VLP group than in the inactivated DENV3 group. The maximum titer was 1:32 in DENV4-VLP group and 1:8 in inactivated DENV4 group. In summary, the maximum neutralizing antibody titer was the highest in DENV2-VLP group and titers were higher in groups that received VLP than in groups that received inactivated virions, except in the case of DENV3 where titers in the VLP and inactivated groups were the same.

**Figure 6:**
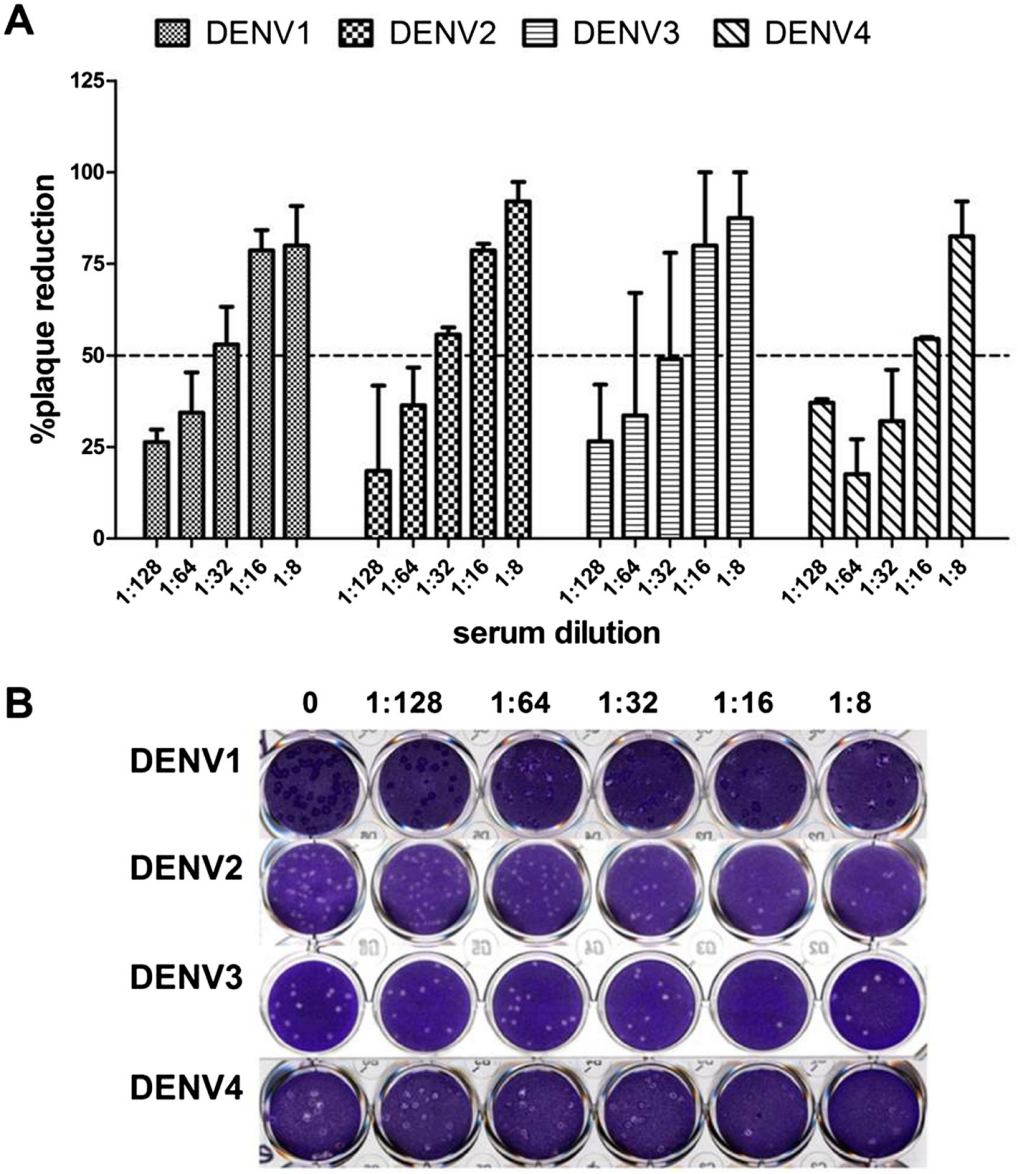
**Detection of tetravalent immune serum neutralizing antibody against DENV.** Balb/c mice were immunized with 100 μg tetravalent DENV-VLP (total of 25 μg for each DENV-VLP serotype) a total of three times at a two week interval. Neutralizing antibodies against DENV1-4 were assessed seven days after the 3rd immunization, using the PRNT_50_ assay. 150-200PFU DENV were incubated with serially diluted mouse antisera in 24-well plates, using BHK-21 cell lines. Data from each group is expressed as the mean percentage of plaque reduction with an SD bar (n = 3). The PRNT_50_ titer of the tetravalent immune sera against the four DENV serotypes were 1:32, 1:32, 1:16, and 1:16, respectively. **(A)** the PRNT50 titer of tetravalent immune serum; **(B)** the plaque morphology.

PRNT_50_ titers induced by tetravalent VLP immunization against each viral serotype were determined in order to demonstrate that monovalent VLP could be combined to produce an effective tetravalent formulation that elicits neutralizing antibodies against all four dengue serotypes. Tetravalent VLP stimulated neutralizing antibodies against all four serotypes (1:32 against DENV1 and 2 and 1:16 against DENV3 and 4) (Figure 6). The highest titers were a little lower than those induced by immunization with the corresponding monovalent VLP.

### Protection of suckling mice from viral challenge

**Figure 7:**
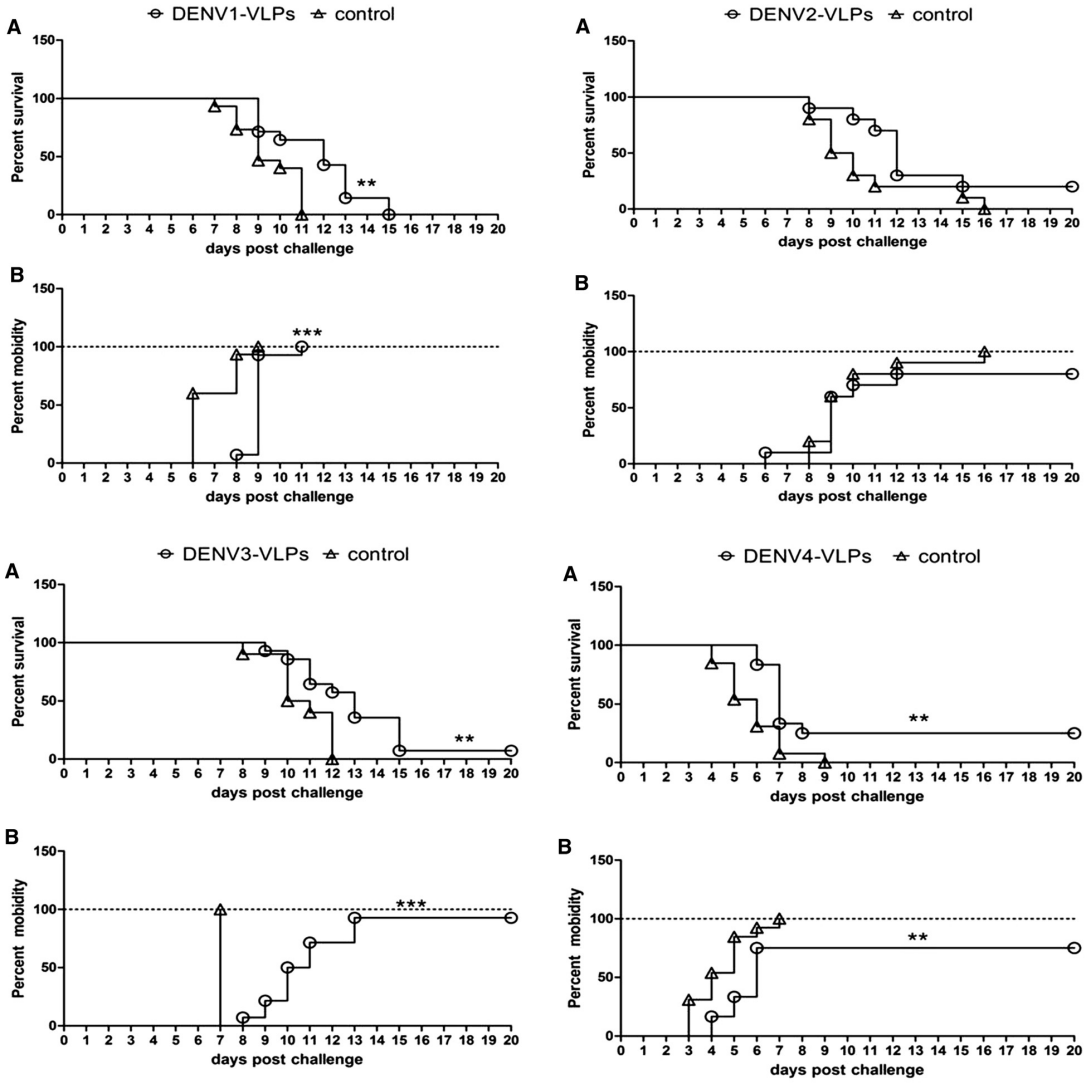
**Protective assay in suckling mice.** Protective effects of monovalent mouse antisera were evaluated in suckling mice. Suckling mice were intracerebrally inoculated with DENV1, 2, 3, or 4, and clinical signs of infection, mainly hind leg paralysis, alterations in spinal column and mortality, were monitored for 21 days. **(A)** survival curve; **(B)** mobidity curve. Different letters mean statistically significant differences by the Log-rank test. *indicates statistical significance (*P < 0.05; **P < 0.01).

Protection induced by monovalent or tetravalent dengue VLPs, in virally challenged suckling mice, was also tested. Control mice challenged with DENV1 began to get sick on day 6, some died on day 7 and all died by day 11. Mice that received anti-DENV1-VLP sera began to get sick on day 8, some died on day 10 and all died by day 15. The differences in morbidity and survival rates were statistically significant between the 2 groups. Control mice challenged with DENV2 began to get sick and die on day 8 and all died by day 11, while the mice that received anti-DENV2-VLP sera began to get sick on day 6, some died on day 8 and two mice (2/10) were still alive on day 21. However, the differences in morbidity and survival rates were not significant between these 2 groups. Control mice challenged with DENV3 began to get sick on day 7, some died on day 8 and all died by day 12, while mice that received anti-DENV3-VLP sera began to get sick on day 8, some died on day 9 and one mouse (1/14) was still alive on day 21. The differences in morbidity and survival rates were statistically significant between these 2 groups. Control mice challenged with DENV4 began to get sick on day 3, some died on day 4 and all died by day 9, while mice that received anti-DENV4-VLP sera began to get sick on day 4, some died on day 6 and three mice (3/12) were still alive on day 21. The differences in morbidity and survival rates were statistically significant between these 2 groups (Figure 7).

**Figure 8:**
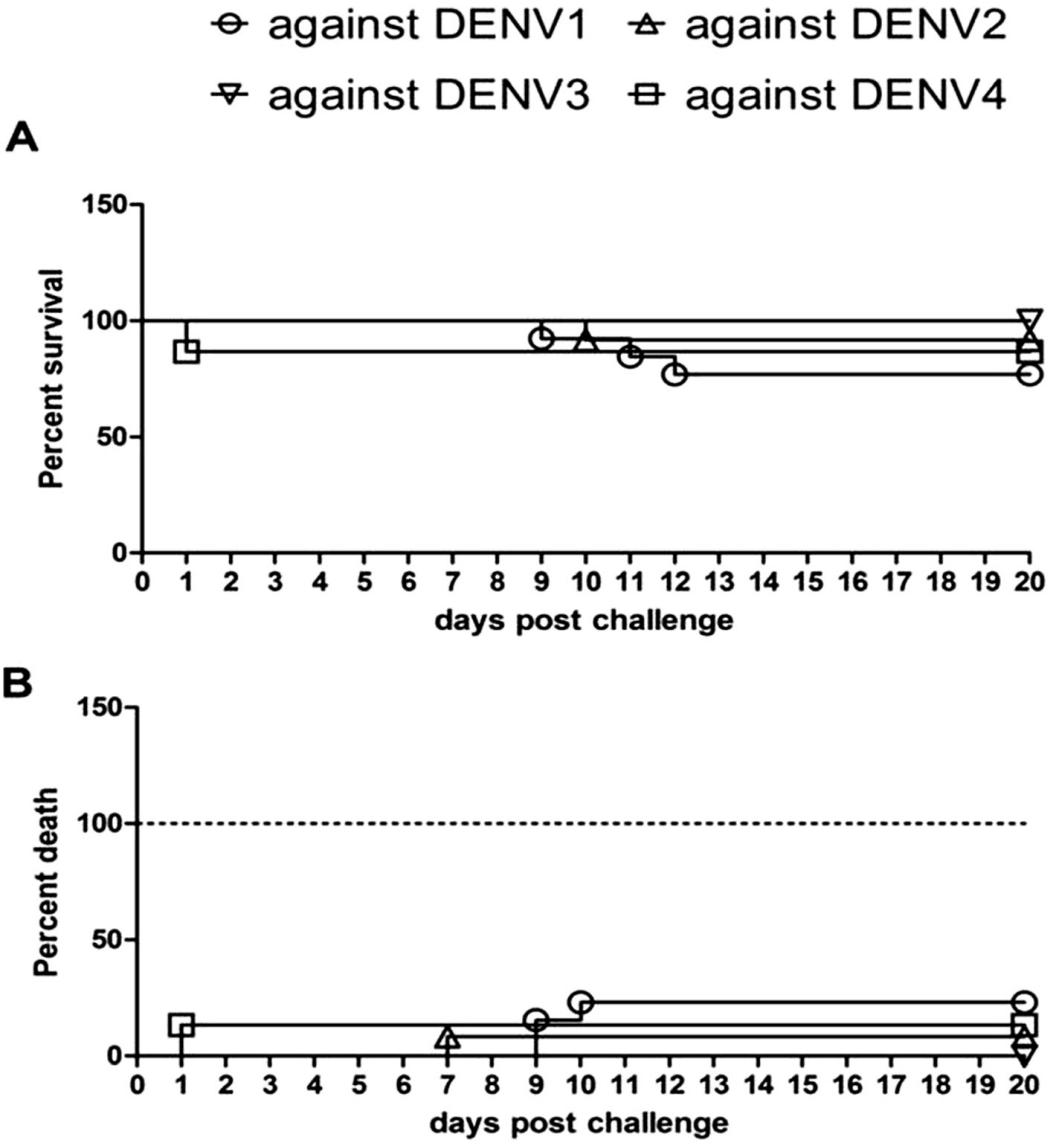
**Anti-tetravalent VLP sera protective assay in suckling mice.** Protective effects of mouse antisera from the tetravalent VLP group were evaluated in suckling mice. Mice were intracerebrally inoculated with DENV1, 2, 3 or 4 and clinical signs of infection, mainly hind leg paralysis, alterations in spinal column and mortality, were monitored for 21 days. **(A)** survival curve; **(B)** mobidity curve. Different letters mean statistically significant differences by the Log-rank test. *indicates statistical significance (*P < 0.05; ** P < 0.01).

Mice in the anti-tetravalent-VLP sera group that were challenged with DENV1 began to get sick on day 9, and some died on day 10, however there were still 10 mice (10/13) alive on day 21. Mice in the anti-tetravalent-VLP sera group that were challenged with DENV2 began to get sick on day 7, and some died on day 10, however there were still 11 mice (11/12) alive on day 21. None of the mice in the anti-tetravalent-VLP sera group got sick or died by day 21 after DENV3 or DENV4 challenge. Although 2 mice in the anti-tetravalent-VLP sera group that were challenged with DENV4 died on day 1, there were no mice got sick or died until day 21. Considering the 2 mice were accidental die, the protection rates against the four DENV serotypes were 77, 92, 100, and 100%, respectively (Figure 8).

## Discussion

VLPs have similar structural and physicochemical features to infectious virions, however they are non-infectious and have advantages in terms of safety and manufacturing. An important characteristic of VLPs is that they can elicit strong humoral and cellular immune responses against viruses [33-35]. Recombinant flaviviruses VLPs have been shown to be able to be efficiently produced by co-expression of prM and E proteins, either with or without C proteins [36,37]. Our previous study demonstrated that DENV-VLP could be successfully expressed in *Pichia pastoris*, based on co-expression of prM and E proteins [30-32]. In that study, it was shown that vaccination with DENV-VLP efficiently elicited virus-specific humoral and cellular immune responses in BALB/c mice. In the current study, the immunogenicity and protective effect of a vaccine made up of four DENV-VLP serotypes was evaluated.

The analysis of humoral immune responses revealed that each of the four dengue VLP serotypes induced antigen specific IgG, similar to the corresponding inactivated dengue virions. The antibody levels were higher in DENV1 and 2-VLP vaccinated groups than in the DENV3 and 4-VLP groups and in the tetravalent VLP vaccinated group than in monovalent groups. Indirect immunofluorescence assay results showed that the antisera from the dengue VLP vaccinated groups could bind with natural dengue virus antigen. In addition, cross-reactions were found between the immune sera from the four DENV-VLP serotypes and the natural DENV virions (data not shown). The results suggest that joint immunization with polyvalent antigens may have a synergistic effect in stimulation of the antibody production.

VLPs not only induce antibody production, but they also have the ability to induce cellular immunity [38,39]. In the present study, cellular immune responses were assessed by the ability of VLPs-stimulated spleen cells to release IFN-γ, TNF-α, and IL-10 cytokines. The number of TNF-α and IL-10 secreting splenocytes was significantly higher in mice that received tetravalent DENV-VLP compared to PBS controls. However, neither IFN-γ nor TNF-α secreting splenocytes were significantly higher in the monovalent DENV-VLP groups (data not shown). IFN-γ is a type of Th1 cytokine, that has an anti-viral effect and also enhances TNF-α production by DENV-infected cells. In addition, high systemic levels of TNF-α have been shown to cause capillary leakage [40]. Serum levels of TNF-α and its receptors have been shown to be elevated in DENV infection and to correlate with DENV disease severity [41,42]. In particular, it has been suggested that IL-10, a Th2 cytokine, to suppress type I IFN-mediated antiviral activity [43]. In addition to the above three cytokines, many other cytokine/chemokines are considered to be correlated with dengue disease severity, including IL-1 β, IL-2, IL-6, IL-8, IL-10, IL-13, IL-18, IFN-γ, TNF-α, and MCP1 [42,44-50]. Results of the current study are not enough to evaluate Th1 or Th2 immune responses produced by tetravalent DENV-VLP or the corresponding dynamic variation. Further studies of T-cell responses are needed to understand the immune status of mice immunized with the tetravalent DENV-VLP.

The protective efficacy of tetravalent DENV-VLP was demonstrated with an antibody neutralization assay *in vitro* and in suckling mice *in vivo*. PRNT_50_ results showed that immunization with either monovalent or tetravalent DENV-VLP induced neutralization antibodies against dengue virus. The PRNT_50_ titers from immune sera of mice immunized with DENV1 and 2-VLP were higher than titers from immune sera of mice immunized with DENV3 and 4-VLP; however, these titers were all higher than immune sera titers reported after immunization with the tandem DENV1-4 domain III of the E protein [51] and higher than the DENV1-4 VLP level expressed in 293 T cell immune sera [52]. The DENV-VLP in the present study showed good immunogenicity and neutralization efficacy, though there are still some differences in efficacy among the four DENV serotypes. The PRNT_50_ titers of immune sera from mice immunized with tetravalent DENV-VLP were a little lower than those from mice immunized with the corresponding monovalent DENV-VLP. Since the antibody levels detected by ELISA and IFA were also higher in the tetravalent VLP group than in the monovalent groups, we deduced that there was no significant relationship between antibody level and neutralization efficacy, particularly since more non-neutralizing antibody against a certain dengue serotype might exist in the tetravalent DENV-VLP immune sera.

The protective efficacy of the tetravalent DENV-VLP vaccine candidate was further tested in a suckling mouse model. The results showed that immunization with either monovalent or tetravalent DENV1-4 VLPs conferred at least some protection, in suckling mice, against DENV1-4 challenge. Furthermore, although the neutralizing antibody level detected was a little lower in the tetravalent formula of DENV1-4 VLP *in vitro*, the tetravalent formula provided more protective efficacy *in vivo* than did the monovalent vaccination. This suggests that the *in vivo* immune response is more complex, and that *in vivo* protective efficacy might be dependent on various immunologic mechanisms and not just the neutralizing antibody. Moreover, the tetravalent formula had better protective efficacy against DENV3 and 4 compared to DENV1 or 2, suggesting that further study of the compatibility of tetravalent DENV1-4 VLP is needed.

## Conclusions

In conclusion, the construction of a tetravalent DENV1-4 VLP vaccine candidate, based on *Pichia pastoris*, is described. The tetravalent DENV-VLP induced humoral immune responses against all four dengue virus serotypes as measured by ELISA or IFA and also induced cellular immunity. The antibody levels were higher, though the neutralizing titers were somewhat lower with tetravalent than with monovalent immunization. Moreover, protective efficacy in the suckling mouse model was better with the tetravalent than with the monovalent vaccination. Further study is needed to adjust the compatibility of the tetravalent DENV1-4 VLP formula and to clarify the amount of cellular immunity that is induced.
